# Supplementary material for: Candida albicans as a Trailblazer for Herpes Simplex Virus-2 Infection Against an In Vitro Reconstituted Human Vaginal Epithelium
Source: Microorganisms. 2025 Apr 14;13(4):905. doi: 10.3390/microorganisms13040905 (PMC12029243; doi:10.3390/microorganisms13040905)
Supplement: Supplementary file 1 [file microorganisms-13-00905-s001.zip › microorganisms-3529360-supplementary.pdf]

Table S1. Summary of events occurring in the RVE model in the presence and in the absence of SVF.

Parameters assessed	Role of the SVF	
Epithelial cell differentiation<br>(IHC staining for cytokeratin 5/6)	↑↑ staining intensity and membrane localization	
Mucin-1 release<br>(ELISA)	↑↑ basal levels	
	**Single infection**	**Double infection**
Candida albicans growth<br>(CFU assay)	↑↑	↑↑
HSV-2 load<br>(DNA copies)	↓	↑
Epithelial cell damage<br>(LDH release)	↑↑ by *Candida albicans*<br>No change by HSV-2	↑↑ by *Candida albicans*<br>No change by HSV-2
Oxidative stress<br>(mtROS)	↑ by *Candida albicans*<br>No change by HSV-2	↑↑ by *Candida albicans*<br>↑↑ by HSV-2
IL-1α production<br>(ELISA)	↑ by *Candida albicans*<br>↓ by HSV-2	↑↑ by *Candida albicans*<br>↔ by HSV-2
IL-1β production<br>(ELISA)	↑ by *Candida albicans*<br>↔ by HSV-2	↑↑ by *Candida albicans*<br>↔ by HSV-2
IL-8 production<br>(ELISA)	↑↑ by *Candida albicans*<br>↔ by HSV-2	↑ by *Candida albicans*<br>↔ by HSV-2
Mucin-1 production<br>(ELISA)	↔ by *Candida albicans*<br>↑ by HSV-2	↓ by *Candida albicans*<br>↓ by HSV-2

Table S1 provides a schematic summary of the results obtained, visualizing the differences between A-431 cells, exposed or not to SVF, regarding *C. albicans* proliferation, virus load, epithelial cell damage, IL-1 α, IL-1 β, and mucin-1 production. We have shown that the SVF promotes A-431 cell differentiation and exerts a pro- *C. albicans* role. The SVF impairs viral replication, that, in contrast, is enhanced in the presence of *C. albicans*. Epithelial cell damage, ROS production and cytokine response are essentially ascribed to *C. albicans*, and, mostly, in the presence of the SVF. Whether these in vitro phenomena may have an in vivo counterpart remains to be investigated. In any case, the RVE may be proposed as a useful model to assess in vitro the complex interplay between vaginal epithelium and single or multiple pathogens.

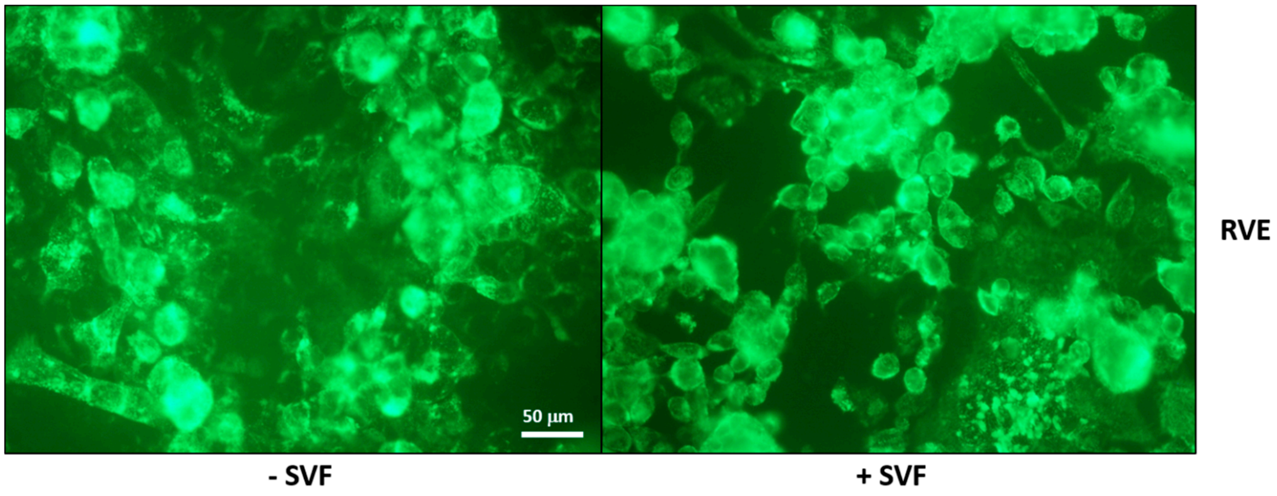

Figure S1- Immunofluorescence assay for HSV-2 detection in RVE cultured with or without SVF.

A432 cells grown for 5 days on chamber slides were infected with HSV-2 (virus:cell ratio = 0.1:1). After 24h incubation, the slides were then fixed in acetone for 15 min. at room temperature and then incubated with a monoclonal antibody against HSV-2 capsid antigen for 35 min. After 3 washes with PBS, a goat anti mouse IgG antibody labelled with FITC was added for 35 min and, following 3 washes with PBS, the slides were counterstained with Evan's blu.

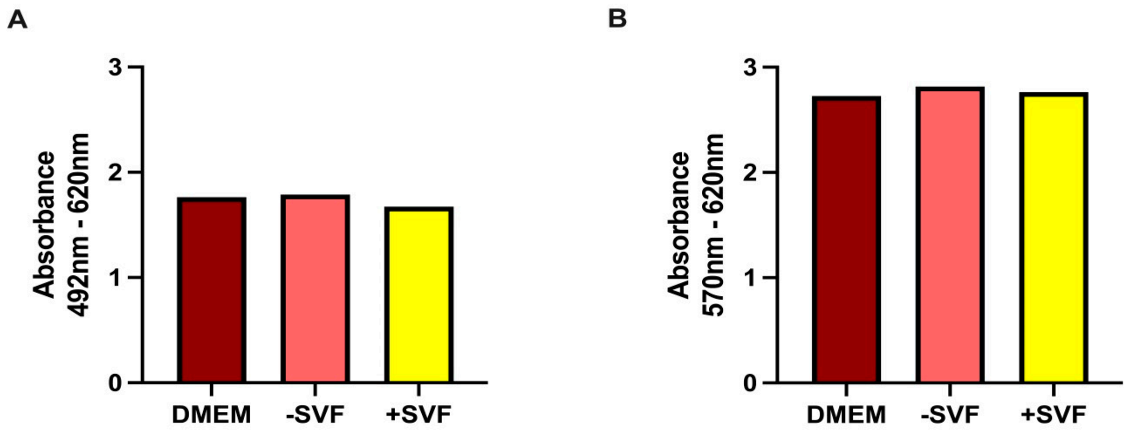

Figure S2 Comparison between LDH and MTT assays for the evaluation of cell damage in RVE cultured with or without SVF.

Five day RVE cultures were incubated with or without SVF for further 24h and then the two cytotoxicity assays were carried out in parallel. The results are completely superimposable.
